# Combined Effect of Melatonin and Sulfur on Alleviating Waterlogging Stress in Rapeseed

**DOI:** 10.1002/pei3.70050

**Published:** 2025-03-27

**Authors:** Md Arif Hussain, Britta Pitann, Karl Hermann Mühling

**Affiliations:** ^1^ Institute of Plant Nutrition and Soil Science Kiel University Kiel Germany

**Keywords:** ascorbate, glutathione, photosynthesis, reactive oxygen species, stomatal conductance

## Abstract

Melatonin, a multifunctional, non‐toxic regulatory molecule, plays a crucial role in enhancing tolerance to abiotic stress, which is tightly linked to S metabolism. Despite the proven efficacy of sulfur (S) in enhancing abiotic stress tolerance, the combined effect of S and melatonin in stress mitigation remains to be elucidated. This is particularly relevant in the context of climate change, where the increased occurrence of waterlogging stress increases the risk of reduced S availability, leading to reduced yield and quality in rapeseed. The objective of this study is to examine the impact of a combination of foliar melatonin and sulfur, when administered to soil or leaves, on the response of plants to waterlogging stress. The experimental design involved the supplementation of rapeseed ( *Brassica napus* L.) plants with sulfur (S) to either the soil (0.2 g kg^−1^) or the leaves (300 ppm) 5 days prior to stress induction. The plants were subjected to waterlogging at BBCH–31 for a period of 7 days, preceded by a pretreatment 2 days prior to the stress with melatonin (200 μM). In comparison, untreated plants subjected to waterlogging showed a significant reduction in growth, nutrient uptake, photosynthetic activity, and sugar content but an increase in the antioxidant defense system. However, the application of melatonin significantly mitigated the adverse effects of waterlogging stress. In comparison with the control, soil‐S application exhibited higher efficacy than foliar S application in increasing plant resistance, as reflected by improved dry weight (+50%), photosynthesis (+12%), stomatal conductance (+40%), sulfur (+40%), magnesium (+59%), and reduced hydrogen peroxide (−22%) and lipid peroxidase (−26%). This combination also increased antioxidant defense by increasing catalase (+43%), glutathione reductase (+17%), ascorbate peroxidase (+47%), ascorbate (+39%), and glutathione (+40%) contents, in contrast to untreated waterlogged plants. The study underlines the potential of melatonin and sulfur as effective agents to alleviate waterlogging stress.

## 1 Introduction

Waterlogging is a significant environmental constraint that restricts agricultural production on a global scale (Aderonmu 2015). Current estimates indicate that approximately 17 million km^2^, constituting 10%–12% of the world's arable land, are at risk of flooding and subsequent waterlogging (Kaur et al. 2019). In Europe, prolonged periods of heavy rainfall during the winter and early spring months have become increasingly prevalent, as evidenced by studies such as that of Deumlich and Gericke (2020). Consequently, Germany has experienced a substantial increase in the frequency of intense precipitation events over the past two decades, a situation that is further amplified in areas where soil quality is suboptimal. These alterations in weather patterns have significant implications for agriculture, given their correlation with substantial yield losses in major crops (Ploschuk et al. 2018; Winterrath et al. 2017). Rapeseed ( *Brassica napus* L.) is particularly susceptible to waterlogging, which can result in yield losses of up to 50% (Hong et al. 2024; Li et al. 2023; Wollmer et al. 2017). Additionally, waterlogging can lead to a decline in the quality of rapeseed, as it increases the levels of glucosinolates and erucic acid content (Xu et al. 2014). It has been demonstrated that this is a significant matter, given that rapeseed is considered to be one of the most important oil crops worldwide. This renders it a pivotal source of oil within the food industry. In addition, rapeseed oilcake, a byproduct of the oil extraction process, serves as a valuable protein supplement in livestock feeding (Hussain et al. 2016).

Waterlogging induces several physiological changes in plants and, by this, affects plant metabolism and growth (Horchani et al. 2009). The main cause of the observed reaction is the induction of a hypoxic/anoxic state within the soil matrix and within the plant root (Armstrong 1980; Ponnamperuma 1972) under waterlogged conditions. Excessive soil moisture has been demonstrated to promote conditions conducive to the loss of nutrients through processes such as leaching (e.g., sulfate and nitrate; Hussain et al. 2022) and denitrification. Furthermore, prolonged soil saturation has been shown to result in oxygen depletion, thereby impacting soil chemistry and the plant rhizosphere. Anoxic conditions induce a shift in soil redox potential (Eh) and pH toward more reducing and acidic states, potentially resulting in nutrient deficiencies or toxicities for plants (Pitann et al. 2025; Wollmer et al. 2017, 2019). For instance, the reduction in Eh induces a series of redox reactions that affect the chemical forms and availability of several micro‐ and macronutrients. While nitrate may undergo rapid reduction, manganese and iron compounds become more soluble, potentially reaching toxic levels for plants (Pitann et al. 2025). Furthermore, under strongly reducing conditions, sulfate may be converted to sulfide, which can be toxic to plant roots. The complex chemical transformations occurring within the rhizosphere, therefore, impose considerable challenges to the survival and growth of plants under waterlogged conditions. Consequently, plants subjected to waterlogging are, for example, at high risk of inadequate S availability.

In planta, oxygen depletion also triggers a rapid shift from aerobic respiration to fermentation, which produces significantly less ATP (Pan et al. 2021). This metabolic change prompts plants to close their stomata, resulting in decreased transpiration. Reduced transpiration impairs water uptake and movement within the plant, consequently limiting nutrient absorption and transport (Colmer and Voesenek 2009; McDonald 2021). The restricted gas exchange at the stomata further hinders CO_2_ uptake, leading to a decline in photosynthetic activity (Anee et al. 2019; Hussain et al. 2022). Moreover, root hypoxia/anoxia results in the generation of reactive oxygen species (ROS) (Hasanuzzaman et al. 2017; Hussain et al. 2024; Ren et al. 2016), a phenomenon that causes photooxidative impairment of diverse cellular processes promoting, e.g., protein degradation, lipoperoxidation, and enzyme deactivation (Nadarajah 2020; Pan et al. 2021). Such processes, combined with the impaired nutrient uptake, significantly decrease plant biomass production and overall yield (Colmer and Greenway 2010; Shao et al. 2013; Voesenek and Sasidharan 2013). However, according to Shabala et al. (2014), the reduction of plant growth via low‐oxygen quiescence may be conceptualized as an immediate random strategy to ensure survival until waterlogging recedes. To counteract the adverse effects of waterlogging stress more targeted, plants have evolved diverse adaptation strategies to sustain their metabolic processes under conditions of oxygen deficiency. These include, among other factors, the formation of aerenchyma, as observed in wheat, maize, barley, and oat (Liang et al. 2020; Pang et al. 2007; Setter and Waters 2003). This adaptation ensures elevated oxygen levels in the root system, enabling the roots to sustain aerobic respiration and, consequently, enhanced nutrient absorption capabilities (Colmer and Greenway 2010). However, due to rapeseed's inability to form aerenchyma, rendering it particularly susceptible to waterlogging (Pang et al. 2007; Voesenek et al. 1999), alternative agronomic adaptive strategies must be considered to mitigate yield losses.

Recently, the role of melatonin (*N*‐acetyl‐5‐methoxytryptamine) has emerged as a significant plant regulator with multifaceted roles in stress resilience, including waterlogging stress (Hasan et al. 2018). This amphiphilic molecule easily crosses cell membranes, acting as a potent antioxidant and regulator of growth, biological rhythms, and stress responses (Arnao and Hernández‐Ruiz 2019; Sun et al. 2021). As such, melatonin regulates key metabolic pathways in plants, e.g., sucrose metabolism (Gao et al. 2022), and plays a protective role against various biotic and abiotic stresses (Khosravi et al. 2023; Moustafa‐Farag et al. 2020), including drought (Liang et al. 2018), oxidative stress (Wang et al. 2015), cold (Bajwa et al. 2014), and heavy metal toxicity (Sami et al. 2020), as well as waterlogging stress (Huo et al. 2022). Furthermore, it is discussed to enhance nutrient uptake (N, S, P) and utilization under stress conditions (Liang et al. 2018; Qiao et al. 2019), and improve nutrient uptake and ion homeostasis to support plant growth (Arnao and Hernández‐Ruiz 2019; Sun et al. 2021). In addition, melatonin enhances sulfur uptake and redox homeostasis, increasing the efficiency of sulfur utilization under low‐sulfur stress (Hasan et al. 2018).

In addition to melatonin, sulfur and its derivatives play a critical role in protecting plants from abiotic stresses by supporting metabolic stability, cellular acclimation, and antioxidant defense (Cao et al. 2014; Hussain et al. 2023; Noctor 2006). For example, sulfur (S) is essential for glutathione (GSH) synthesis, which is affected by sulfur availability under stress conditions such as waterlogging in rapeseed (Hussain et al. 2023). Sulfur supplementation, either by soil or foliar application, increases plant survival by improving stress adaptability and metabolic processes. In addition, sulfur improves antioxidant systems and nutrient assimilation to mitigate the effects of abiotic stress (Anjum et al. 2015; Hasanuzzaman et al. 2017; Hussain et al. 2024; Waraich et al. 2022).

Although the sole effects of either melatonin or sulfur supplementation under abiotic stress have already been investigated, studies on the combined effect, particularly under waterlogging stress in rapeseed, are still scarce. Furthermore, the effectiveness of these treatments often relies on the form of application, with differences in soil and foliar application, as demonstrated for sulfur by Hussain et al. (2024). Consequently, the present study aims to examine the combined effect of melatonin and sulfur on growth parameters, as well as physiological and nutritional responses. The central hypothesis of this study is that the combination of melatonin and sulfur (S) supplementation will enhance waterlogging resistance.

## 2 Materials and Methods

### 2.1 Plant Material and Plant Cultivation

Seeds of rapeseed ( *Brassica napus* L. cv. Campino; Norddeutsche Pflanzenzucht Lembke, Germany) were soaked overnight and subsequently planted in pots containing1.5 kg of loamy sand (pH_CaCl2_ 6.5; sand: 85%, silt: 10.9%, clay: 4.1%; S: 6.5 mg kg^−1^ soil). Basal fertilization was executed as explained by Hussain et al. (2023). At the vegetative stage BBCH–31, waterlogging treatments were implemented by maintaining a water level of approximately 3 cm above the soil surface for a period of seven consecutive days. It is crucial to note that all pots designated for the control plants were meticulously maintained at a uniform soil moisture level of 80% WHC (water holding capacity) throughout the duration of the experiment. Sulfur supplementation (S) was applied to the soil (0. 2 g kg^−1^ soil) or to the leaves (300 ppm) as MgSO_4_·7H_2_O, as outlined in a previous study (Hussain et al. 2024), 5 days prior to the waterlogging treatment. Additionally, 200 μM melatonin (MT) was sprayed equally to soil‐S‐ and foliar‐S‐treated plants 2 days prior to waterlogging treatments. In a preceding screening process, the optimal melatonin dose was ascertained for a concentration range of 50–350 μM, with the objective of precluding any potential damage arising from leaf burn.

Approximately 15 mL of foliar spray (S and MT) with 0.1% Silwet Gold as a wetting agent was applied once in the evening, ensuring that lower temperatures were maintained, preventing evaporation of the sprays. An equivalent volume of deionized water (dH_2_O) was applied to the control plants, ensuring the maintenance of comparability. During the spraying process, the pots were covered with aluminum foil to prevent carryover to the soil. The plants were cultivated under controlled greenhouse conditions, with relative humidity of 62%–70%, a photoperiod of 14/10 h (day/night), a day/night temperature of 22/18°C, and a light intensity of 350 μM photon m^−2^ s^−1^ (recorded with Li–198 light meter, Lincoln, NE, USA). The experiment was organized by a completely randomized design (CRD), taking four treatments along with four replications per treatment.

The treatments were arranged as follows:

1. Control (well‐watered); C
2. Waterlogging; WL
3. Waterlogging + Soil‐S + Foliar Melatonin (WL + SS + FM)
4. Waterlogging + Foliar S + Foliar Melatonin (WL + FS + FM)

### 2.2 Morphological Assessment and Sampling

Prior to harvesting 7 days after the implementation of the waterlogging treatment, plant height was recorded. In this experiment, half of the plant material from each treatment was harvested and stored in liquid nitrogen at −80°C for subsequent analysis. The remaining plants were cut at the base, and their fresh weights were recorded. Additionally, dry weights from the plants were determined after drying at 65°C for 72 h. To ensure comprehensive analysis, all dried samples were milled to a fine powder (MM 200, Retsch GmbH, Germany) for further analysis.

### 2.3 Gas Exchange Measurement

The net photosynthetic rate and stomatal conductance were assessed at the seventh day of waterlogging prior to harvesting for all treatments. An infrared gas analyzer (IRGA) system (Li‐Cor 6400; Li‐Cor, Lincoln, NE, USA) was employed in this experiment, as described by Amoako et al. (2024). The topmost fully expanded leaf from each treatment was assessed from 9:00 a.m. to 11:00 a.m. on a core 6‐cm^2^ leaf segment. The air temperature was fixed at 25°C and the intensity of CO_2_ was set at 400 μmol mol^−1^ with CO_2_ addition in the reference chamber. Finally, net photosynthesis (Pn) was expressed in μmol CO_2_ m^−2^ s^−1^ and stomatal conductance (gs) by mol m^2^ s^−1^.

### 2.4 Determination of Nutrient Contents

Leaf sulfur content was determined using the Dumas combustion method (ICC 167). Briefly, a powder sample of 5–10 g was weighed together with the equal volume of wolfram‐(IV)‐oxide into a tin capsule. The total S concentration was subsequently determined using an elemental analyzer (Flash EA 1112 NCS, Thermo Fisher Scientific, USA). To ensure standardization and recognition of the reference material, a sample of wheat flour (IVA 33802156; IVA Analysis Technology GmbH & Co., Germany) was incorporated into each batch.

To assess mineral nutrient content, 200 mg of dried leaf powder was processed with 10 mL of 69% HNO_3_ (ROTIPURAN Supra for ICP, 69%) in an 1800‐W microwave oven (MARS 6 Xpress; CEM, Matthews, MC, USA) for 45 min. Subsequently, all samples were attenuated with deionized water (18.2‐MΩ cm conductivity) to 100 mL, and were then stored at 4°C until analysis. The concentrations of nutrients were computed by using ICP‐OES (inductively coupled plasma‐optical emission spectroscopy; Agilent −5800, Böblingen, Germany).

### 2.5 Determination of H_2_O_2_ and Malondialdehyde Content (MDA)

Frozen rapeseed leaves were utilized for the estimation of H_2_O_2_ and malondialdehyde content (MDA). The H_2_O_2_ level was assessed as explained by Hussain et al. (2023) with minor modifications. In detail, 0.3 g of frozen sample was blended in a cold bath with 1.6 mL of 0.1% TCA (trichloroacetic acid). Subsequently, the mixture was centrifuged at 13,000 *g* at 4°C for 25 min. Afterward, 500 μL of the resulting supernatant was transferred into 500 μL of 100 mM K‐P buffer (pH 7.8), together with 1 mL of 1 M potassium iodide (KI). The mixture was then briefly vortexed and incubated in the absence of light for 1 h at room temperature. Subsequently, the absorbance of H_2_O_2_ was photometrically measured at 390 nm. The results were estimated from an H_2_O_2_ calibration curve, and the contents were expressed as μmol g^−1^ fresh weight (FW).

For the estimation of lipid peroxidation, the thiobarbituric acid (TBA) test was used to calculate malondialdehyde (MDA) content, as described by Heath and Packer (1968). Briefly, 0.25 g of chilled leaf powder was blended in 1 mL of 5% TCA (w/v) solution in a cold mortar. Afterwards, the mixture was centrifuged at 12,500 *g* for 15 min. Thereafter, 1 mL of supernatant was transferred into 4 mL of 0.5% (w/v) TBA in 20% TCA. Subsequently, the sample combinations were immersed in warm water at 95°C for 30 min, followed by cooling in an ice bath. Following this, the samples were centrifuged at 12,500 *g* for 5 min, and the absorbance was recorded at 532 nm. To account for non‐specific absorption at 600 nm, this value was then subtracted from the total. The amount of MDA content was estimated by utilizing an extinction coefficient of 155 mM^−1^ cm^−1^, and the value was expressed as nmol g^−1^ FW.

### 2.6 Extraction and Measurement of Ascorbate and Glutathione Content

For the determination of glutathione and ascorbate contents, 500 mg of frozen samples were crushed in liquid nitrogen and blended with 4 mL of 5% (w/v) TCA as described by Hussain et al. (2023). Then the homogenate underwent centrifugation for 15 min at 12,000 *g* at 4°C. The resultant supernatants were accumulated for glutathione (GSH) and ascorbate (AsA) determination.

The calculation of total ascorbate necessitated the use of a neutralized solution, which was achieved by the application of 0.2 mL of the supernatant into 0.3 mL of K‐P buffer (0.5 M; pH 7.0) together with 0.1 mL of DTT (0.1 M), thereby reducing the oxidized portion. Conversely, to neutralize reduced ascorbate (AsA) solution, an equal quantity of dH_2_O was used in place of 0.1 M DTT. Following the vortexing step, all samples were stored at room temperature for 40 min. The reaction assay was then designed by using K‐P buffer (pH 6.5) and ascorbate oxidase, in addition to the neutralized solution. Subsequently, a change in the degree of absorbance at 265 nm was measured in the presence of 0.5 units of ascorbate oxidase (AO). A standard curve of known concentrations of ascorbate was prepared in advance and used to calculate the total ascorbate and ascorbate (AsA) concentrations. Afterward, dehydroascorbic acid (DHA) was calculated by subtracting ascorbate (AsA) from total ascorbate. Finally, the content of AsA was presented as nmol g^−1^ FW.

Oxidized glutathione and total glutathione content were assessed as reported by Hussain et al. (2023) with minor modifications. Briefly, the total GSH content was determined by neutralizing aliquots with 0.5 M K‐P buffer (pH 7.0) and oxidizing with DTNB (5,5′‐dithio‐bis‐(2‐nitrobenzoic acid)) in the presence of glutathione reductase (GR; baker yeast, type III, Sigma–Aldrich, USA) and NADPH (reduced nicotinamide adenine dinucleotide phosphate). Subsequently, the absorbances were documented at 412 nm. To estimate oxidized glutathione (GSSG), 2‐vinylpyridine was added and left at room temperature for 1 h to neutralize the extract along with 0.5 M K‐P buffer for 1 h at room temperature. The concentration of total GSH and GSSG was then estimated by applying a standard curve of known concentrations. Finally, the GSH content was calculated by subtracting the values of GSSG from the total GSH. The content of GSH was expressed in μmol g^−1^ FW.

### 2.7 Antioxidant Enzymes Estimation

#### 2.7.1 Extraction of Crude Plant Extract

To collect crude plant extracts, all steps were performed at 4°C. Subsequently, approx. 400 mg of ice‐cold leaf powder was homogenized in 1 mL K‐P buffer (50 mM; pH 7.0), containing 1 mM AsA, 5 mM β‐mercaptoethanol, 100 mM KCl together with 10% glycerol (w/v). Thereafter, the crude plant extracts were centrifuged at 12,000 *g* at 4°C for 15 min. The upper layer liquid was then stored for estimating enzyme activities. However, the spectrophotometric calculations were performed using a spectrophotometer (UV–vis; Thermo Helios Gamma, UK).

#### 2.7.2 Total Proteins

Total soluble proteins were measured in accordance with the Bradford (1976) method. Bovine serum albumin (BSA) solution was used as a protein standard, and its absorbance was computed at 595 nm.

#### 2.7.3 Assay of Antioxidant Enzymes

Catalase (CAT; EC: 1.11.1.6) activity was calculated following Aebi (1984), with slight modifications as reported by Hussain et al. (2023). The activity of CAT was computed by spotting the decline of H_2_O_2_ in the presence of plant extract. Then, the reaction was initiated by combining K‐P buffer (50 mM; pH 7.0), 15 mM H_2_O_2_, and the plant extract, yielding a final volume of 0.7 mL. Subsequently, the change in absorbance was measured at 240 nm for the duration of 1 min at room temperature. Finally, the extinction coefficient of 0.039 mM^−1^ cm^−1^ was implemented to evaluate CAT activity, with a single unit of enzyme being exhibited as 1 μM of the substrate that reacted per mg protein per minute.

Ascorbate peroxidase (APX; EC: 1.11.1.11) activity was determined using the method of Nakano and Asada (1981), as described by Mohsin et al. (2019). However, the APX activity was derived from AsA‐dependent reduction of H_2_O_2_. The test combination comprised plant extract, 0.1 mM H_2_O_2_, 0.1 mM EDTA, 0.5 mM AsA, and 50 mM K‐P buffer (pH 7.0), with a total volume of 0.7 mL. Subsequently, the initiation of the reaction was marked by the introduction of H_2_O_2_, and the absorbance was conducted for a duration of 1 min at 290 nm. Finally, the extinction coefficient of 2.8 mM^−1^ cm^−1^ was used to estimate the APX activity and presented as μmol of H_2_O_2_ min^−1^ mg^−1^ protein.

Glutathione reductase (GR; EC: 1.6.4.2) activity was computed in accordance with the method reported by Manzoor et al. (2023). Briefly, the oxidation of glutathione‐dependent NADPH was detected at 340 nm. Subsequently, the response combination was constructed with 0.1 M K‐P buffer (pH 7.8), 1 mM GSSG, 1 mM EDTA, 0.2 mM NADPH, along with extract from the plant in an ultimate quantity of 1 mL. Finally, the response was initiated by supplementing GSSG, and the decline in light absorbance was examined for 1 min. Afterwards, the GR activity was determined by employing 6.2 mM^−1^ cm^−1^ (extinction coefficient) and displayed as nmol min^−1^ mg^−1^ protein.

### 2.8 Statistical Analysis

The experiment was conducted with four replications in a completely randomized design (CRD). Data were interpreted based on two‐way ANOVA by applying R (version 4.3.1). Factors were aligned by adopting Pearson correlation analysis. At the 5% probability level, Tukey's HSD test was used to estimate variances among the studied parameters, and the data were displayed as the means ± standard error (SE). The graphs were prepared using GraphPad Prism 8 (version 8.2.1).

## 3 Results

### 3.1 Effect of MT and S on Plant Growth and Net Photosynthesis Under Waterlogging

**FIGURE 1:**
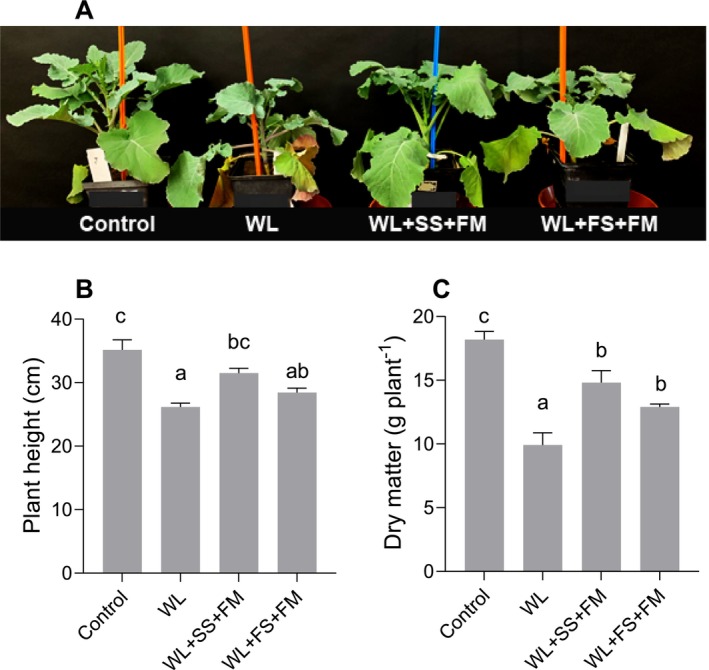
Effect of foliar melatonin combined with soil or foliar‐applied S on morphological alteration (A), plant height (B), dry matter (C) of waterlogged (WL), soil sulfur (SS), foliar melatonin (FM), and control *Brassica napus* plants. The data are means ± SE of four independent pot replicates. The bars followed by different letters indicate significant differences between the treatment groups (Tukey's test; *p* ≤ 0.05).

The objective of this study was to investigate the role of external melatonin supply in combination with sulfur in mitigating the adverse effect of waterlogging stress on rapeseed. For this, melatonin was applied in combination with sulfur either to soil or leaves. Plants subjected to 7 days of waterlogging showed severe growth inhibitions, as indicated by reduced plant height, dry matter, leaf chlorosis, and lodging (Figure 1). In contrast, the application of soil‐S and foliar melatonin to waterlogged plants resulted in a significant increase in plant height and dry matter (Figure 1). Likewise, plants that received foliar sulfur and melatonin showed a slight increase in plant height, along with a significant increase in dry matter, when compared with untreated waterlogged plants. In addition, the stomatal conductivity and photosynthesis rate of waterlogged plants were significantly lower than those of the corresponding control (Figure 3). However, the plant that received soil‐S and foliar melatonin showed a significant increase in both stomatal conductivity and photosynthesis rate in contrast to untreated waterlogged plants. As shown in Figure 3, foliar application of S and melatonin resulted in a modest enhancement in stomatal conductivity and photosynthesis rate, with increases of 12% and 40%, respectively, in comparison with untreated waterlogged plants.

### 3.2 Effect of MT and S on Waterlogging‐Induced Oxidative Stress

To elucidate the effectiveness of a combination of exogenous melatonin and foliar/soil‐S as a promising tool to mitigate waterlogging‐induced oxidative stress, the focus was directed towards ROS aggregation and membrane lipid peroxidation in rapeseed. The results showed that the levels of MDA and H_2_O_2_ were significantly higher under waterlogging conditions (Figure 4). However, this accumulation was significantly reduced by the application of melatonin in combination with soil‐S or foliar S. Interestingly, the decrease of H_2_O_2_ and MDA in melatonin‐supplemented plants treated with soil‐S was more pronounced (by 22% and 26%, respectively) than in plants supplemented with foliar S under waterlogging conditions (Figure 4).

### 3.3 Effect of MT and S on Antioxidant Defense System in Rapeseed Under Waterlogging

To understand the relationship between the combined treatment of exogenous melatonin and foliar/soil‐S treatment on ROS and lipid peroxidation retardation and the alterations in antioxidant enzymes and metabolites, CAT, GR, and APX activities as well as GSH and AsA contents in the leaves of rapeseed were measured (Figure 5). While CAT and GR activity were not significantly altered under waterlogging conditions when compared to control plants, APX clearly responded to the stress condition, showing a significant increase (Figure 5A–C). However, the combination of melatonin and soil‐S supply resulted in the highest CAT, APX, and GR activities overall. Conversely, foliar S supplementation proved to be less or even non‐effective in comparison to the soil‐S treatment, particularly for CAT and APX (Figure 5A,B), while a similar efficacy as for soil‐S on GR was observed (Figure 5C).

With regard to GSH/GSSG and AsA as antioxidative active molecules, a similar pattern can be observed. In the same way that GSH significantly decreased under waterlogging conditions as compared to the control, GSSG increased (Figure 5D,E). Similar to the antioxidative enzymes, the combination of foliar S with melatonin was less effective in comparison to soil‐S‐treated plants, being only significant in the case of GSSG (Figure 5D,E). Waterlogging stress also resulted in a significant increase in AsA compared to the control group. This increase was further augmented when melatonin was applied in combination with soil‐S (Figure 5F). Conversely, foliar S supplementation showed the lowest efficacy among all S and melatonin treatments, although it was significantly higher than in untreated plants under waterlogging (Figure 5F).

### 3.4 Effect of MT and S on Nutrient Uptake

**FIGURE 2:**
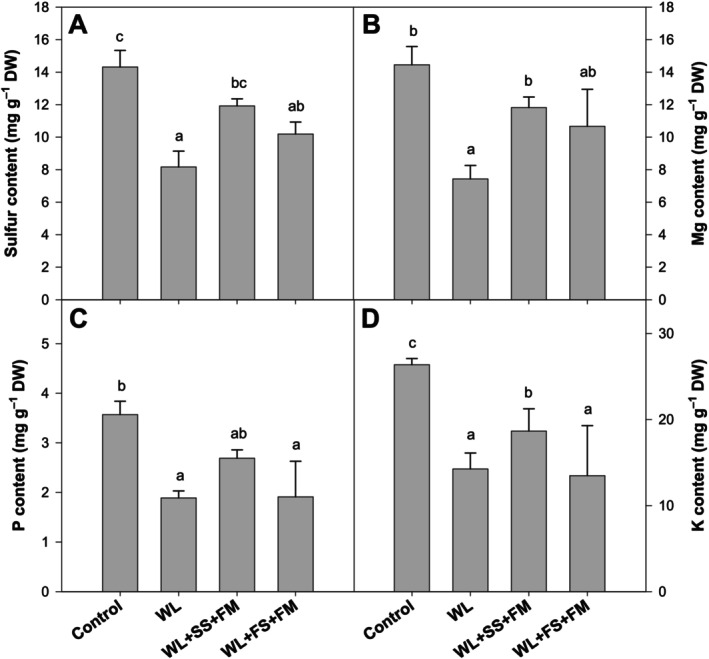
Effect of foliar melatonin combined with soil or foliar‐applied S on S, Mg, P, and K in leaves of *Brassica napus* grown under waterlogged and control conditions. The data are means ± SE of four independent pot replicates. The bars followed by different letters indicate significant differences between the treatment groups (Tukey's test; *p* ≤ 0.05).

Waterlogging resulted in a reduction of the macronutrients S, Mg, P, and K when compared to the non‐stressed control plants (Figure 2). When melatonin was supplemented in combination with sulfur, an increase of all nutrients was observed. This increase, however, was only significant in the case of soil S application, with concentrations reaching the level of the corresponding control (Figure 2).

## 4 Discussion

The primary factors influencing crop yield are climate‐related, including solar radiation, temperature, and precipitation. However, human‐induced climate change is amplifying these factors, thereby increasing the probability of extreme weather events (Rosenzweig et al. 2001). A salient example is the occurrence of excessive rainfall, which can result in waterlogging, a condition that substantially diminishes crop yields. For instance, waterlogging has been linked to annual yield reductions of up to 50% and nutritional quality in key crops like wheat, maize, rapeseed, rice, and soybeans (Borgomeo et al. 2020; Ding et al. 2020; Hong et al. 2024; Ploschuk et al. 2018; Tian et al. 2021; Wollmer et al. 2017). The identification and breeding of crop species exhibiting tolerance to waterlogging, in combination with the application of agronomical practices, have the potential to mitigate the adverse effects on crop physiology and enhance agricultural resilience (Kaur et al. 2020).

### 4.1 Combined MT and S Application Can Improve the Nutritional Status of Rapeseed

Sulfur‐associated characteristics have been identified as crucial in determining the yield and quality of crops. However, despite the central focus on sulfur‐related traits in research, advancements remain relatively modest. This, on the one hand, underscores the intricate nature of sulfur regulatory networks, but, on the other hand, also highlights the pressing need for further investigation into sulfur utilization and metabolism, particularly under abiotic stress (Koprivova and Kopriva 2016). However, S has already been identified as a pivotal element in enhancing plant resilience against various abiotic stresses, including waterlogging (Bouranis and Chorianopoulou 2023; Hussain et al. 2023, 2024). Low S availability, which is the case under waterlogging, has been demonstrated to result in severe growth retardation and stress susceptibility even after a short time (Hasan et al. 2018; Hasanuzzaman et al. 2019; Siddiqui et al. 2019). This is in line with the results of this study (Figure 2), where low S availability and thus uptake can be assumed to be the reason for the substantial decline in growth (Figure 1) under waterlogging. These results are further consistent with the findings of other studies conducted on rapeseed (Hussain et al. 2024; Zhou et al. 1997), wheat (Wollmer et al. 2018), oat (Arduini et al. 2019), and maize (Ren et al. 2016). However, as demonstrated by Hussain et al. (2023, 2024), S supplementation via the root and/or the leaves constitutes a viable strategy to mitigate the deleterious effects of waterlogging on the growth of rapeseed. This phenomenon can be attributed not only to the replenishment of the internal S pool but also to the synergistic relationship that facilitates the uptake of other major nutrients (Shah et al. 2022; Sharma et al. 2024).

In the past few years also research on phytomelatonin has progressed, indicating its potential to protect plants against oxidative stress (Tan et al. 2014). Drought‐induced ROS stress was thereby effectively reduced by MT‐induced higher APX, CAT, and SOD activities (Campos et al. 2019). To date, a substantial body of research has emerged demonstrating the efficacy of exogenous melatonin application in enhancing abiotic stress tolerance (e.g., Ahmad et al. 2023; Hassan et al. 2022; Zhang et al. 2021; Zhang et al. 2024; Zeng et al. 2022). Especially when it comes to stress‐related S deficiency in plants, as is the case under waterlogging, MT application has emerged as a promising new tool because of the direct link between melatonin and sulfur metabolism (Hasan et al. 2018; Marques et al. 2024). For example, Marques et al. (2024) showed that melatonin application promoted growth and productivity of soybean, which was directly associated with an increase in S absorption and metabolic activity after melatonin application. However, data on the relevance of the combined effect of melatonin and S, particularly on waterlogging stress resistance, are scarce and remain to be elucidated.

In this study, a clear effect of a combined S and melatonin application on plant growth and selected physiological parameters was found. Similarly to the individual application of S (Hussain et al. 2024), plant height and dry weight were significantly improved (Figure 1). However, the application of soil S was found to be more efficacious in mitigating stress compared to foliar S application, which is in line with Hussain et al. (2024). Such a difference is attributable to the enhanced sulfate uptake capacity via the root, but the limited absorption potential via the leaf. As sulfate (SO_4_^2−^) is the main form of sulfur available to plants in soil, the root uptake of this form is favored and facilitated by high‐affinity transporters regulated by *SULT1:1* and *SULT1:2* (Leustek 2002). In conditions where S is limited, as can also be assumed for waterlogging conditions, MT application has been observed to slightly augment sulfate concentrations in comparison to treatments lacking MT (Marques et al. 2024). This effect was even more pronounced under optimal sulfur application compared to the low S treatment. Furthermore, MT has been observed to positively influence sulfur translocation in soybean leaves (Marques et al. 2024), both in the presence and absence of sulfur. This may also explain the higher S content in soil‐S‐treated plants compared to the foliar application when combined with MT (Figure 2). Similarly, Hasan et al. (2018) described the distinct role of MT in S acquisition and assimilation in tomato plants under low‐sulfur stress. Moreover, Hasan et al. (2019) observed that MT enhanced S uptake and assimilation in *Solanum lycopersicum* under cadmium (Cd) stress by upregulating sulfate transporter genes (*SUT*). Similar results were also described for other nutrients that become limited under abiotic stress, such as drought. Liang et al. (2018) demonstrated that external MT enhanced N uptake by increasing the transcripts of the relevant genes (*NRT*; *AMT*) in apple trees. The findings of this study are further substantiated by the conclusions of Zhang et al. (2017), who found that MT plays a substantial role in regulating the nutrient composition of cucumber in response to nitrate stress. Consequently, it is reasonable to assume that this effect is attributed to the potential role of MT in activating genes involved in nutrient uptake and translocation, which are often repressed under stress conditions (Takahashi 2019). This may also explain the improved in planta nutrient status (Mg, P, K) in this study upon MT application (Figure 2), with the more pronounced effect under soil compared to foliar S application. However, the effect of MT on the regulation of transporters and uptake efficiencies under waterlogging needs to be elucidated in the future.

### 4.2 Combined MT and S Application Positively Affects Photosynthesis

Waterlogging is not only affecting the nutritional status of the plant, but also various metabolic processes, which eventually lead to growth inhibition (Horchani et al. 2009; Hussain et al. 2024; Iqrar and Abdin 2017). In order to cope with these challenging conditions, plants have been observed to increase their endogenous melatonin level (Zheng et al. 2017), which is initiated by a post‐transcriptional process further driven by the uptake of exogenous melatonin. However, the process of adaptation to abiotic conditions such as waterlogging also involves a complex crosstalk between MT and other phytohormones (e.g., auxin, abscisic acid, ethylene; Khan et al. 2022), as well as other signaling metabolites (nitric oxide (NO)).

**FIGURE 3:**
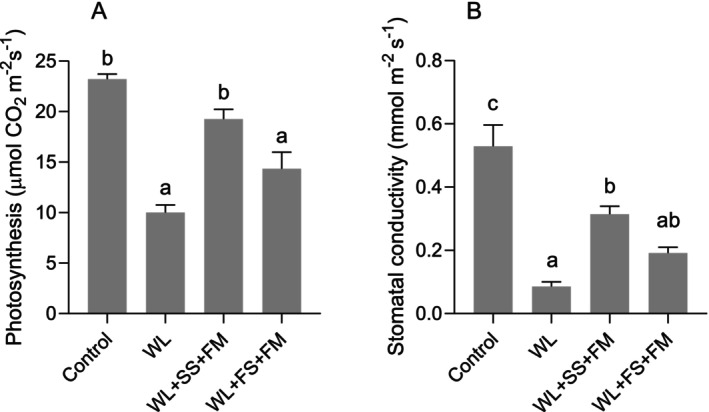
Effect of foliar melatonin combined with soil or foliar‐applied S on photosynthesis rate (A), stomatal conductivity (B) of waterlogged (WL), soil sulfur (SS), foliar melatonin (FM), and control *Brassica napus* plants. The data are means ± SE of four independent pot replicates. The bars followed by different letters indicate significant differences between the treatment groups (Tukey's test; *p* ≤ 0.05).

Waterlogging‐induced growth inhibition (Figure 1) can also be attributed to a decline in photosynthesis (Figure 3) resulting from a hampered gas exchange at the stomata due to reduced stomatal conductance and thus CO_2_ uptake (Shao et al. 2013). This is in line with previous studies (e.g., Hussain et al. 2024), which clearly indicated a reduction in net photosynthesis and stomatal conductivity under waterlogging in rapeseed. Reduced photosynthesis together with a lack of internal S often gives rise to a metabolic imbalance of S‐containing compounds and subsequent oxidative stress in plant cells (Carciochi et al. 2017). This overproduction of ROS, primarily H_2_O_2_, in turn causes photooxidative damage to chloroplasts (Hasanuzzaman et al. 2017; Ren et al. 2016; Zhang et al. 2015). Thus, waterlogged plants typically exhibit symptoms such as chlorosis, particularly in the older basal leaves (Arbona et al. 2008), which were also present in this experiment (Figure 1) and have also been described by Wollmer et al. (2018) for wheat.

MT has been demonstrated to exert a protective effect on the plant photosynthetic apparatus, for example in tomatoes (Liu et al. 2015) and maize (Ye et al. 2016). Similarly, the improvement of photosynthesis and stomatal conductivity can be particularly attributed to the application of MT in apple and cucumber (Li et al. 2015; Zhang et al. 2017). This effect was even boosted when MT and S were applied (Marques et al. 2024), which was also evident from this study (Figure 3). Preventing the collapse of chloroplasts from oxidative damage is imperative, as chloroplasts are also the site of MT synthesis. Thus, adequate S and MT are necessary for maintaining chloroplast structure and function and thereby protecting photosynthesis (e.g., Waraich et al. 2022; Wu et al. 2020; Zhang et al. 2019).

### 4.3 Combined MT and S Application Boosts Antioxidative Defense

It is well known that both S and MT play a significant role in the effective scavenging of ROS by inducing several enzymes associated with the antioxidative defense system in plants, thereby mitigating the detrimental effect of waterlogging (Arnao and Hernández‐Ruiz 2019; Doupis et al. 2017; Hasanuzzaman et al. 2018).

In accordance with prior research, it was shown that particularly soil‐S supplementation plays a pivotal role in mitigating waterlogging stress by enhancing antioxidant enzymes and metabolites (e.g., GSH) in rapeseed. GSH is recognized as a pivotal antioxidant and free radical scavenger (Hussain et al. 2023). For example, Chandra and Pandey (2014) reported that metabolic inequality of GSH significantly provokes oxidative burst in *Allium* sp. under low S‐stress. In addition, in stressed plants, the requirement of GSH increases to improve stress resistance through the stimulation of enzymes, such as SOD, CAT, APX, and GR, engaged in the antioxidant defense, as already shown for rapeseed (Hussain et al. 2024). As a consequence, the improved net photosynthesis and stomatal conductance of rapeseed are ascribed to the external application of S (Hussain et al. 2024), underscoring the pivotal function of S in stress alleviation.

**FIGURE 4:**
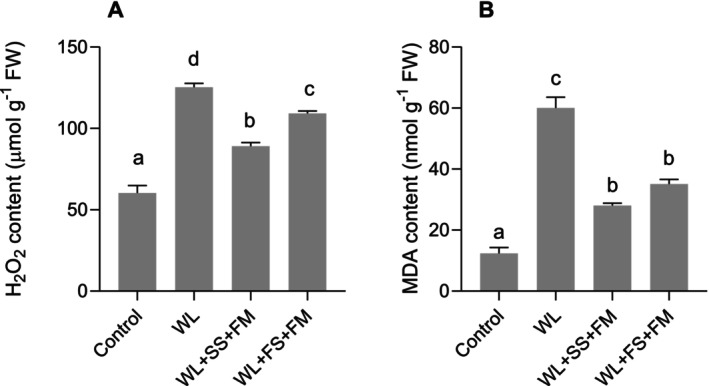
Effect of foliar melatonin combined with soil or foliar‐applied S on H_2_O_2_ contents (A) and MDA contents (B) of waterlogged (WL), soil sulfur (SS), foliar melatonin (FM), and control *Brassica napus* plants. The data are means ± SE of four independent pot replicates. The bars followed by different letters indicate significant differences between the treatment groups (Tukey's test; *p* ≤ 0.05).

**FIGURE 5:**
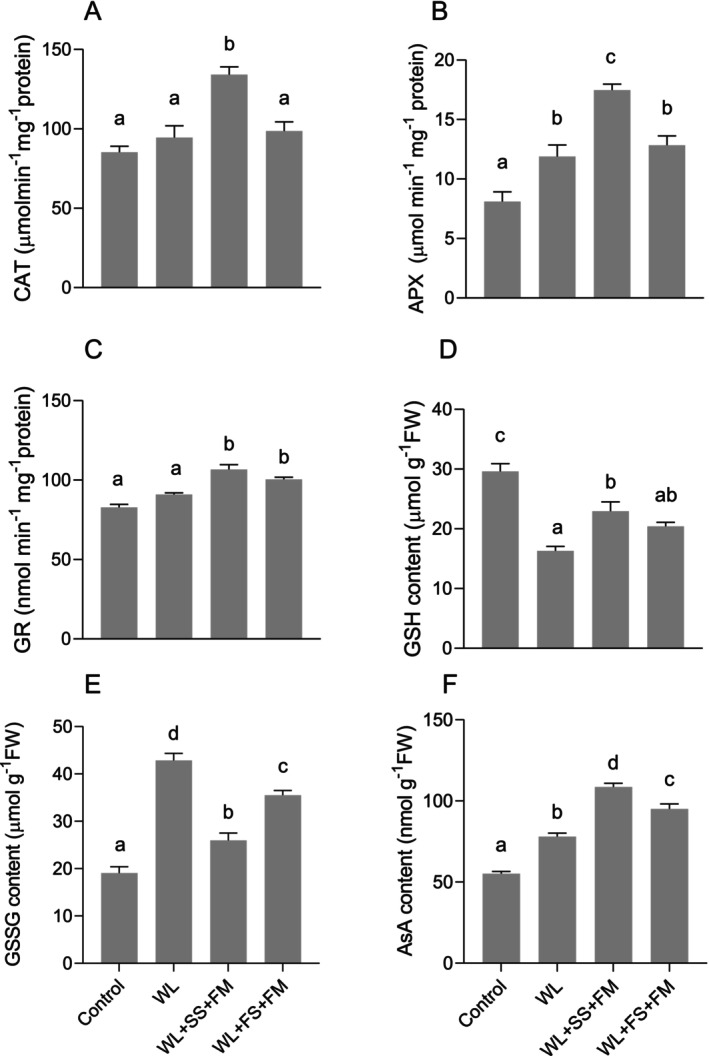
Effect of foliar melatonin combined with soil or foliar‐applied S on catalase (CAT) activity (A), ascorbate peroxidase (APX) activity (B), glutathione reductase (GR) activity (C), glutathione (GSH) content (D), oxidized glutathione (GSSG) content (E), and ascorbate (AsA) content (F) of waterlogged (WL), soil sulfur (SS), foliar melatonin (FM), and control *Brassica napus* plants. The data are means ± SE of four independent pot replicates. The bars followed by different letters indicate significant differences between the treatment group (Tukey's test; *p* ≤ 0.05).

Similarly, under waterlogging also MT application was shown to be effective in upregulating the activity of the antioxidative enzymes SOD, POD, and CAT in apple seedlings subjected to waterlogging for 9 days (Zheng et al. 2017). By this, elevated levels of H_2_O_2_ and O_2_^·−^, caused by waterlogging stress, were significantly reduced and which was also indicated by a reduced MDA level. Also, Ahmad et al. (2022) reported that foliar spraying of MT in maize alleviated the waterlogging stress through upregulating the antioxidant system. The combination effect of S + MT is well documented in the literature; however, there is negligible focus on crop plants. For example, Haghi et al. (2022) reported the synergistic effect of MT and S on alleviating lead toxicity in safflower. This effect was a result of improved phytochelatin content and the activity of enzymes of the ASC‐GSH cycle. These findings corroborate the results of the present investigation, in which it was shown that S supplementation, whether to soil or leaves in combination with MT, reduces oxidative stress markers (H_2_O_2_) and lipid peroxidation (MDA) (Figures 3 and 4). The reduced levels of ROS and MDA can be attributed to the activation of the enzymatic antioxidative defense machinery (Hasanuzzaman et al. 2019). Not only were enzymes such as CAT, APX, and GR increased in their activity, but also GSH and AsA contents were significantly higher under combined S and MT treatment (Figure 5).

### 4.4 Soil‐S Application Is Superior to Foliar S Application When Combined With MT

The results of this study clearly demonstrate that soil‐S supplementation is superior to foliar S supplementation when combined with foliar MT. It can be inferred that plants have a preference for SO_4_^2−^ uptake from soil, which may be attributed to increased transporter expression (e.g., *SULT1*, *SUT*; Hasan et al. 2018; Leustek 2002), in the presence of MT (cf. Section 4.1). While rapeseed is known not to produce aerenchyma to circumvent O_2_ depletion and thus hampered water and nutrient uptake (Voesenek et al. 1999), it can be hypothesized that MT application also had a positive effect on root architecture. Although not measured in this study, other findings report a positive effect of MT. Liang et al. (2017), for instance, demonstrated that MT application modulated the auxin response in rice, which ultimately increased embryonic root length and the number of crown roots. Similarly, root biomass was significantly increased upon MT application under drought conditions in tomato (Altaf et al. 2022) and rapeseed (Dai et al. 2020). MT application has also been shown to be effective in pumpkin, increasing not only root length but also root vitality under waterlogging stress (Liu et al. 2024). Therefore, it is reasonable to assume that MT application would also be effective in the root architecture of rapeseed, which would explain the better efficacy of soil‐applied S compared to the foliar S treatment.

Although the preliminary experiment ruled out the negative effects of a sole MT spray (data not shown), visual observations revealed that the combined application of foliar melatonin and foliar sulfur (S) induced slight scorching and burning effects on the plant leaves (Figure 1). One possible reason for the observed symptoms can be explained by the frequent foliar application of both substances with 2‐day intervals, possibly increasing liquid density and affecting plant health (Bouranis and Chorianopoulou 2023; Phillips and Mullins 2004; Linzon et al. 1979). Moreover, the selection of the optimal growth stage has the potential to enhance S utilization efficiency post‐foliar application. For instance, the efficacy of foliar sprays was maximized after choosing an early application timepoint (Woolfolk et al. 2002). Similarly, the incidence of foliar burn from mineral nutrients can undergo a 70% reduction by targeting an earlier growth stage (Girma et al. 2007). However, in view of this finding, it is imperative to make precise dosage adjustments prior to implementing MT and S in field conditions to avert phytotoxicity.

## 5 Conclusion

To date, the involvement of melatonin and sulfur in diverse physiological and biochemical processes under waterlogging in rapeseed plants remains largely unknown. Waterlogging impeded nutrient homeostasis and plant growth by interfering with various physiological traits related to stress‐induced ROS formation. This results in extreme levels of oxidative impairment, as shown by elevated hydrogen peroxide (H_2_O_2_) and malondialdehyde (MDA) in the leaves of rapeseed. The application of S, though, whether foliar or soil‐based, in combination with MT has been shown to alleviate waterlogging stress by reducing H_2_O_2_ and MDA levels, enhancing in planta nutrient levels, and promoting parameters such as net photosynthesis, stomatal conductance, and antioxidative defense. However, the combination of MT and soil‐S led to a more pronounced resistance compared to MT and foliar S supplementation in waterlogged rapeseed plants. These findings already provide substantial evidence that MT and S act synergistically to boost resistance to waterlogging stress in rapeseed. Nevertheless, future research must focus on understanding the molecular basis that leads to the improved nutrient status, as well as the crosstalk with other stress‐relevant phytohormones.

Despite the preliminary findings indicating enhanced resilience to waterlogging in rapeseed, further investigation is necessary to assess long‐term implications on productivity, also for other crops and varieties. Additionally, the development of cost‐effective, sustainable, and environmentally friendly formulations is crucial.

## Conflicts of Interest

The authors declare no conflicts of interest.

## Data Availability

All data are presented in the article itself.
